# The Impact of Alcohol Restriction on Hospital and Emergency Department Service Utilizations in Two Remote Towns in the Kimberley Region of Western Australia

**DOI:** 10.3389/fpubh.2019.00017

**Published:** 2019-02-26

**Authors:** Wenxing Sun, Le Jian, Jianguo Xiao, Grant Akesson, Peter Somerford

**Affiliations:** ^1^Epidemiology Branch, Department of Health, Government of Western Australia, Perth, WA, Australia; ^2^School of Public Health, Curtin University, Perth, WA, Australia; ^3^Primary Health Tasmania, Hobart, TAS, Australia

**Keywords:** alcohol restriction, alcohol-related hospitalization, injury, domestic violence, emergency department attendance, alcohol-related atiological fractions, interrupted time series analysis, autoregressive integrated moving average

## Abstract

**Background:** In a remote region of Western Australia, Kimberley, residents have nearly twice the State average per capita consumption of alcohol, four and a half times the level of alcohol-related hospitalizations and nearly three times the level of alcohol-related deaths. This study aimed to evaluate the long term effects of alcohol sale restrictions on health service utilization in two remote towns in Kimberley.

**Methods:** Sale of high strength packaged alcohol was restricted in Fitzroy Crossing and Halls Creek since October 2007 and May 2009, respectively. Alcohol-related Emergency Department (ED) attendances and hospitalizations utilized by local residents before and after the intervention between 2003 and 2013 was compared by using yearly rates (/1,000 person-years) and interrupted time series analysis with Autoregressive Integrated Moving Average (ARIMA) modeling. The Western Australia specific aetiological fractions (AAFs) were applied to hospital inpatient data for estimation of the proportion of hospital separations attributable to alcohol.

**Results:** In Fitzroy Crossing, there was a significant reduction of over 40% on rates (/1,000 person-years) of alcohol-related acute hospitalizations (54.2 [95% CI: 53.8–54.7] vs. 31.7 [31.4–32.1]) and ED attendances (534.1[532.8–535.5] vs. 294.5 [293.5–295.4]). In Halls Creek, there was a significant reduction of over 50% on rates (/1,000 person-years) of alcohol- related acute hospitalizations (17.7 [17.6–17.8] vs. 8.0 [7.9–8.1]) and ED attendance (248.4 [247.9–248.9] vs. 111.1[110.8–111.5]). Domestic violence and injury related hospitalization rates were also reduced by over 20% in both towns.

**Conclusions:** The total restriction of selling high strength alcohol through a community driven process has shown to be effective in reducing alcohol-related health service utilization in post-intervention period. Continue monitoring is required to address new emerging issues. Future research on health service utilization related to alcohol by using interrupted time series analysis incorporating ARIMA modeling and applying AAFs are recommended for evaluating alcohol-related interventions.

## Introduction

The misuse of alcohol is a major cause of injuries, domestic violence, chronic diseases and deaths worldwide, accounting for nearly 10% of global deaths among people aged 15–49 years (1). The harmful consequence is often worse among Indigenous population compared to the general population (2–4). In Australia, alcohol alone contributed to 5% of the total disability adjusted life years and one-third of the burden due to road traffic injuries (5). The attributable burden in 2011 due to alcohol and illicit drug use in Indigenous Australians was 4.2 times the rate of non-Indigenous Australians (6). With 26% of Indigenous population, Northern Territory of Australia has the second highest alcohol consumption per capita in the world, estimated 15.1 L of pure alcohol per year (7).

**Figure 1:**
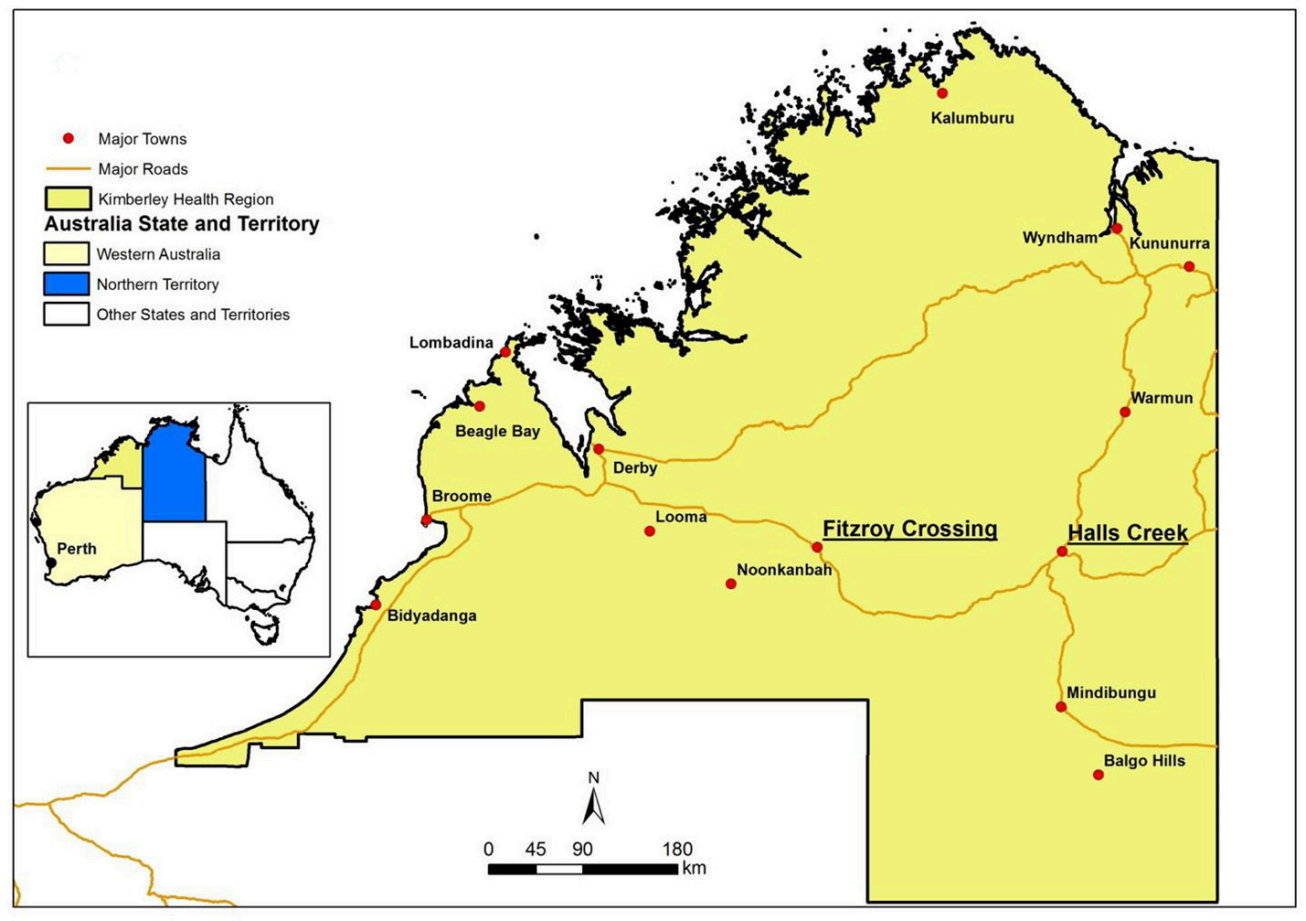
Locations of the two study sites in the Kimberley Health Region of Western Australia.

The Kimberley region, to the west of the Northern Territory (Figure 1), is another area that has a high density of Indigenous residents (45% of Kimberley population). The Kimberley Health Region, defined by the Department of Health Western Australia (WA), is the northernmost region of WA. It is about 2000 km away from Perth (the capital city of WA). The Kimberley region has nearly twice the State average per capita consumption of alcohol, four and a half times the level of alcohol-related hospitalizations and nearly three times the level of alcohol-related deaths (8, 9). Alcohol was a major contributor of emergency department (ED) attendances for injuries and interpersonal violence (10–13). Two major towns in the Kimberley region are Fitzroy Crossing and Halls Creek, both with Indigenous people making up in excess of 90% of the population. It was estimated that the rate of outpatient hospital presentations related to alcohol in these two towns was greater than the rest of Kimberly region (14).

A number of substantial economic literatures documented that restricting access to alcohol reduced alcohol-related harms such as mortality, crime, and injuries (15–17). For Indigenous communities in Australia, the culture, tradition, poverty, homelessness, unemployment, overcrowding and mining royalty make the motivation for engaging in high-risk drinking behavior (18). Although these factors make the interventions difficult to break through, legal restrictions that are specifically designed for local communities have shown to have favorable effect, at least in the short term (19–23). Recent reports described the benefit of the alcohol sale restriction in Fitzroy Crossing (since 2007) and Halls Creek (since 2009) in reducing numbers of alcohol-related ED presentations and police activities (24, 25). The community driven process has been fundamental to its success in the first 2 years (26). However, these studies only reported the number changes and did not take into account population changes over years, and alcohol-related hospitalizations were not reported.

This study examined both hospital inpatient and ED attendances over the 10-year period (2003–2013) using routinely collected data which captured both local admissions as well as those subsequently transferred to hospitals in the State for alcohol-related conditions. Rates were derived instead of counts to adjust the variations related to population change. To our knowledge, this is the first study in Australia that applied WA specific alcohol-related aetiological fractions (AAFs) and used interrupted time series analysis to evaluation the long term impact of the alcohol sale restriction in remote areas.

## Materials and Methods

### Alcohol Restriction Intervention Program

The WA Director of Liquor Licensing imposed alcohol restrictions in the towns of Fitzroy Crossing and Halls Creek in October 2007 and May 2009, respectively, to “prohibit the sale of packaged liquor exceeding an ethanol concentration of greater than 2.7% at 20°C to any person, other than a lodger (as defined in Section Results of the WA Liquor Licensing Act 1988).” The lodge is prohibited from selling alcohol before 12:00 midday, except when accompanied by a meal or sold to a lodger. After 12:00 midday, full-strength alcohol can still be purchased in the hotels (14, 27). The Halls Creek restriction was on an indefinite basis from the implementation; while the Fitzroy Crossing restriction was implemented initially for a period of 6 months and then extended on the 18 May 2008 indefinitely following a review (25).

### Data Sources and Case Ascertainment

**Figure 2:**
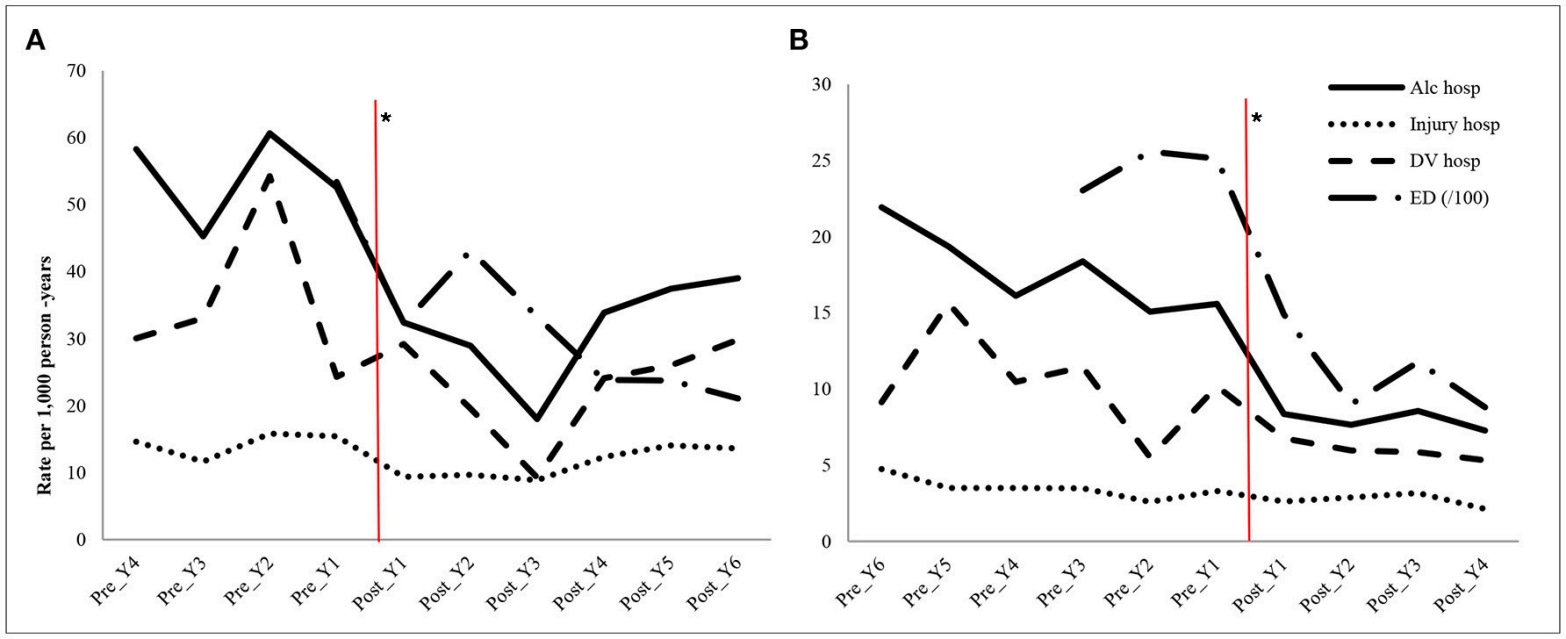
Annual rates (/1000 person-years) of hospitalizations and emergency department (ED) attendances in Fitzroy Crossing **(A)** and Halls Creek **(B)** during pre- and post-intervention periods. ^*^The vertical lines indicating the start of the intervention; Y, year; Alc, alcohol-related acute conditions; hosp, hospitalizations; DV, Domestic violence; ED (/100), the ED rates are per 100 person-years.

Anonymous hospital administrative records associated with Fitzroy Crossing or Halls Creek residents (i.e., with a postcode of 6765 or 6770) from 2003 to 2013 were extracted from WA Hospital Morbidity Data System using International Statistical Classification of Diseases and Related Health Problems, 10th Revision, Australian Modification (ICD-10-AM). During the 10-year study period, the pre- intervention period for Fitzroy Crossing was 4 years (October 2003-September 2007) and the post- intervention period was 6 years (October 2007-September 2013); while it was the opposite for Halls Creek where the pre- (June 2003-May 2009) and post- (June 2004-May 2013) intervention periods were 6 and 4 years, respectively. The intervention years were labeled as pre-Y1, pre-Y2, post-Y1, post-Y2 and so on in Figure 2 with Y1 being the closest to the intervention year. ICD-10-AM codes for alcohol- related acute conditions used in the data extraction included T51 (Toxic effect of alcohol),V02-V04 (Pedestrian injury from motor vehicle), V09 (Unspecified pedestrian injury),V12-V14 (Cyclist injury from motor vehicle), V19-V89 (Transport accident), W00-W19 (Falls), W24-W31 (Mechanical injury), W45 (Foreign body entering through skin), W60 (Injury from plant thorns), W65-W74 (Accidental drowning and submersion), W78-W79 (Aspiration), X00-X09 (Fire injury), X60-X84 (Intentional self-harm), X85-Y09 (Assault), and Y87.1 (Sequelae of assault). Episodes from non-local residents who had treatment in the local hospital were excluded via reviewing their residential postcodes. The total alcohol-related acute conditions were then split to injury related (all codes above except T51, W65-W74, X85-Y09) and domestic violence related (X85-Y09).

The WA specific AAFs representing the proportion of hospital separations attributable to alcohol were applied to hospital inpatient data. The detailed method of deriving these AAFs was described elsewhere (28). In summary, if a condition (such as alcoholic gastritis) is wholly attributable to alcohol, an AAF value of 1 will be applied to that condition; whereas a condition that is partially attributable to alcohol (such as liver cancer), an AAF value of less than 1 will be applied to that condition. These AAFs were derived using the relative risk of the condition in the population and the WA prevalence of alcohol consumption by age, gender and Aboriginality. The former came from systematic meta-analyses of cohort, case-control and cross sectional studies, and the latter was unpublished data specifically obtained from the Australian Bureau of Statistics. A typical formula was used for calculating AAFs for conditions partially attributable to alcohol as below:

(1) $$\begin{array}{r} AAF=\frac{\sum P_{i}\left(RR_{i}-1\right)}{\sum P_{i}\left(RR_{i}-1\right)+1} \end{array}$$

where *Pi* is the prevalence of alcohol consumption at risk level i and *RRi* is the relative risk of the relevant condition associated with alcohol consumption at risk level *i* compared with non-alcohol consumption (28–30) .

WA Emergency Department Data Collection provided ED data for patients with postcodes of 6765 and 6770 for the period of 2006–2013 (ED data is available since 2006). Due to lack of availability of the ICD-10-AM codes in patient record system from remote hospitals, a broad disease category in ED data, major diagnostic categories 20 (alcohol/drug use and alcohol/drug induced organic mental disorders) and 21 (injuries, poisonings, and toxic effects of drugs) were used to best describe alcohol related conditions.

### Study Design and Statistical Analysis

The interrupted time series analysis was carried out on monthly data to test the “interruption” of the intervention to the underlying trend over the 10-year period. Autocorrelation was adjusted using the Autoregressive Integrated Moving Average (ARIMA) models. Dummy variables were included in the models to represent the pre- and post- intervention periods. The appropriateness of the model was assessed by using the Ljung-Box statistic (33). The seasonality was tested using the Augmented Dickey-Fuller method. All series were stationary and lagged differencing was not required (i.e., no *d* component in final ARIMA models). All statistical analyses were performed in SAS version 9.2 (SAS Institute, Cary, NC).

Rates of hospitalizations and ED attendances per 1,000 person-years were calculated to compare the average difference between pre- and post- intervention and yearly measures for the study period. The annual population (person-years) was derived based on the estimated resident population, an Australia's official measure of populations (31). The 95% confidence intervals (32) for the rates were derived using the formula: rate ± 1.96 × standard error, where standard error = $\sqrt{rate/person\text{-}years}$.

## Results

In Fitzroy Crossing, the yearly average number of alcohol-related hospitalizations and ED presentations over the 4 years before the intervention (October 2003–September 2007) were 61 and 615, respectively; the yearly average numbers over the 6 years after the intervention period (October 2007–September 2013) were 38 and 353, respectively. Meanwhile in Halls Creek, the yearly average number of alcohol-related hospitalizations over 6 years before the intervention (June 2003–May 2009) was 67 and that for ED presentations was 933; over the 4 years after the intervention (June 2004–May 2013), the average number per year was 31 for alcohol-related hospitalizations and 433 for ED presentations. In both towns, more females were (>50%) admitted to hospital for alcohol-related conditions than males throughout the study period (Table 1) which was accompanied by approximately 80% of female victims for domestic violence-related hospitalizations. It decreased to 73% in post-intervention period in Halls Creek, but remained steady in Fitzroy Crossing (82%). In terms of ED attendances, there were more females (56%) in Halls Creek in the pre-intervention period, and dropped to 51% in the post-intervention period. The gender variation in Fitzroy Crossing was smaller—females were 2% higher than males throughout the study period.

**Table 1:** Gender and age distribution (%) of Fitzroy Crossing and Halls Creek residents admitted to hospital or emergency department (ED) in the pre- and post-intervention periods.

		**Fitzroy crossing**				**Halls creek**			
		Alcohol hosp^*^	Injury hosp^*^	Domestic violence hosp^*^	**ED**	Alcohol hosp^*^	Injury hosp^*^	Domestic violence hosp^*^	**ED**
Female	Pre%	55.3	50.0	80.6	53.8	55.5	42.3	79.9	56.1
	Post%	57.3	51.1	81.9	52.2	55.5	47.2	73.1	50.7
<20 y	Pre%	13.1	20.8	11.3	28.1	14.0	24.3	18.8	24.9
20–29 y		24.7	24.9	26.3	23.1	33.1	26.0	38.0	25.7
30–39 y		28.6	18.7	33.1	25.5	28.3	20.3	30.8	25.6
>39 y		33.6	35.7	29.4	23.3	24.6	29.5	12.4	23.9
<20 y	Post%	14.7	17.7	20.5	26.8	12.2	16.5	10.8	29.6
20–29 y		28.9	22.5	35.5	28.4	28.4	25.9	33.3	23.8
30–39 y		23.7	22.3	22.3	19.8	24.5	19.3	24.7	21.1
>39 y		32.8	37.5	21.7	25.0	34.9	38.2	31.2	25.6

* *hosp, hospitalizations*.

The age group proportions before and after intervention in both towns were also displayed in Table 1. All alcohol-related hospitalization indicators and ED attendances shown a proportional decrease for people aged 30–39 years in both towns except for injury-related hospitalizations in Fitzroy Crossing where there was a proportional increase.

The average annual alcohol-related hospitalizations per 1,000 person-years reduced in the post-intervention period compared to the pre-intervention period (Table 2) for all indicators in both towns. In Fitzroy Crossing, there was a reduction of over 40% of alcohol-related hospitalizations (54.2 [95% CI: 53.8–54.7] vs. 31.7 [31.4–32.1]) and alcohol-related ED attendances (534.1 [532.8–535.5] vs. 294.5 [293.5–295.4]). The reduction in hospitalizations due to alcohol-related injury and domestic violence were 21 and 35% over 6 years after the intervention, respectively. The interrupted time series analysis confirmed the decline of all measures with p values < 0.05 for alcohol-related hospitalizations, injury, domestic violence and ED attendances. The decline on the rate of ED attendances (13.2 per 1,000) was the greatest among the four measures. In Halls Creek, the intervention resulted in a significant reduction of over 50% of alcohol-related hospitalizations (17.7 [17.6–17.8] vs. 8.0 [7.9–8.1]) and ED attendances (248.4 [247.9–248.9] vs. 111.1[110.8–111.5]). The interrupted time series analysis confirmed the decline of these two measures while there were no statistically significant changes of injury and domestic violence related hospitalizations due to alcohol.

**Table 2:** Average annual rate (/1,000) of pre- and post- intervention and interrupted time series analysis of hospitalization and emergency department (ED) attendances in Fitzroy Crossing and Halls Creek.

	**Measure**	**Average rate pre (95% CI)**	**Average rate post (95%CI)**	**% Decrease**	ARIMA model^*^	**Estimate**	***P*-value**
Fitzroy Crossing	Hospital^#^	54.2 (53.8–54.7)	31.7 (31.4–32.1)	−41.5	(0, 5)	−2.23	<0.01
	Injury	14.4 (14.2–14.6)	11.4 (11.2–11.6)	−20.8	(0, 0)	−0.61	<0.01
	Domestic violence	35.5 (35.1–35.8)	23.1 (22.8–23.4)	−35.0	(0, 3)	−1.45	<0.05
	ED	534.1 (532.8–535.4)	294.5 (293.5–295.4)	−44.9	(1, 0)	−13.18	<0.05
Halls Creek	Hospital^#^	17.7 (17.6–17.8)	8.0 (7.9–8.1)	−55.0	(1, 1)	−0.42	<0.01
	Injury	3.5 (3.5–3.6)	2.7 (2.6–2.8)	−23.2	(0, 0)	0.02	>0.05
	Domestic violence	10.4 (10.3–10.5)	6.0 (6.0–6.1)	−42.4	(2, 0)	−0.08	>0.05
	ED	248.4 (247.9–248.9)	111.11 (110.8–111.4)	−55.3	(1, 0)	−9.33	<0.01

* All series were stationary- no differencing (d) were required.

# *Hospital, hospitalization*.

The yearly trend over the whole study period in Figure 2 has shown that the Fitzroy Crossing had a few noticeable fluctuations. For example, the average rate of alcohol-related hospitalizations was 54.2/1,000 person-years (95% CI 53.8–54.7) before the intervention, reducing to 32.4/1,000 person-years (95% CI 32.1–32.7) 1 year after and rising again non-significantly to 39.0/1,000 person-years (95% CI 38.7–39.4) 6 years later. The rate of alcohol-related ED attendance in post year 2 (year 2009) slightly increased (432.0/1,000 person-years, 95% CI 430.8–433.1) but the rate was still significantly lower than the overall pre intervention period (534.1/1,000 person-years, 95% CI 532.7–535.4). However, the overall hospitalizations for all alcohol-related conditions and ED attendances were still significantly lower than the pre-intervention period.

In Halls Creek, the trend was steadier over the post intervention period. The average rate of alcohol-related hospitalizations was 17.7/1,000 person-years (95% CI 17.6–17.8) before the intervention, reducing to 8.4/1,000 person-years (95% CI 8.3–8.5) 1 year after the intervention and further decreasing to 7.3/1,000 person-years (95% CI 7.2–7.4) 4 years after. The hospitalization rate of alcohol-related domestic violence decreased from 10.2/1,000 in 1 year prior to the intervention to 5.3/1,000 in the fourth year post the intervention. The alcohol-related ED attendance rate was 124.2/1,000 person-years (95%CI 123.8–124.5) in the pre-year period and decreased to 88.1/1,000 person-years (95%CI 87.8–88.4) in the fourth year after the intervention.

## Discussion

This study has demonstrated the positive effect of community driven alcohol sale restriction on health service utilization in Fitzroy Crossing and Halls Creek, two remote towns of WA. We applied WA specific AAFs for the first time to better detect changes of hospitalizations due to alcohol. The 10-year study period provided an overall picture for all indicators and covered a longer evaluation period than previous reports (24, 25). The rates per 1,000 person-years before and after the intervention were derived to adjust results in relation to local population changes. Over the study period, there were clear declining trends of all measured indicators in both towns while the population numbers in both postcodes steadily and slowly increased.

In addition, this study was a retrospective evaluation of an intervention which was implemented at a clear time point by using the interrupted time series analysis. Interrupted time series analysis is widely used in public health intervention studies internationally but very few used in evaluations of alcohol restriction interventions (33–36). This method includes a quasi-experimental design and is considered as the “next best” approach for evaluation of interventions when randomization is not possible, providing the assumptions are met (37). The three basic assumptions for the use of interrupted time series analysis are: the data consist of a linear trend; the characteristics of the study population remain unchanged throughout the study period and no comparator against the intervention itself. In regards to our study, due to small population size of the study areas, there was a fluctuation of outcome measures over time, especially in the Fitzroy Crossing. Nevertheless, the overall trend was linear and the ARIMA modeling was incorporated in the study to correct the autocorrelation of the time series data. Due to the unique location of both towns which are in an extremely remote region with a great proportion of Aboriginal population, we could not find any towns with similar population characteristics as comparators or synthetic controls. Given the fact that the same population was used as the control group (for pre-intervention period) as well as the study group (for post-intervention period), the socio-economic conditions, culture and lifestyle of the population in these two remote areas remain unchanged except the “interruption” of alcohol sale restriction intervention. This is one of the advantages of this study design. Meanwhile, the impact of population number change was taken into account by calculating rates.

While the alcohol sale restriction contributed to a significant reduction of overall alcohol-related hospitalizations and ED attendances in both towns, there were a few non-significant fluctuations cross the whole post intervention period. These fluctuations might partially owe to the small population of the study areas which made the change sensitive. For example, the increase in the ED attendance in 2009 (post year 2) in Fitzroy Crossing might be related to a local incident that happened in the same year. It was described in the WA Drug and Alcohol Office 2010 report (24) that “assaults had increased immediately after the Tarunda Supermarket burned down (on July 9, 2009) because people began traveling to Derby and Broome to purchase food and other items and also full-strength take-away alcohol.” The same report also presented qualitative finding that community members expressed strong support for the continuation of this restriction (24–26). The community driven intervention was a key success of the restriction in Indigenous communities (3, 21, 38).

In comparison to Fitzroy Crossing, the rates of all health service indicators in Halls Creek were lower in both pre- and post-intervention periods. There were some differences in the availability of alcohol and the length of restrictions in place between the two towns which might partly explain this observation. Halls Creek only has one source of alcohol sale outlet and the nearest town with alcohol outlets is located 360 km away in the north (i.e., Kununurra). In addition, Halls Creek had a different alcohol sale restriction since November 1992 (38). Other concurrent community programs such as school education program, the introduction of the Community Development Employment Program, expansion of TAFE services and arts center were also in place to which might contribute to bringing down alcohol consumption in the community. These might partially explain the relative lower alcohol-related health service usage in Halls Creek than Fitzroy Crossing.

There were a few limitations of the study. Firstly, we were not able to apply AAFs to the ED data because the ICD-10-AM diagnostic codes were not assigned in the ED records in remote hospitals. Further improvement on the ED data collection is required to include either the ICD-10-AM codes or an alcohol use status. In order to evaluate alcohol related health service utilization accurately, collecting information on alcohol consumption in administrative data will be extremely useful. The second limitation was related to the lack of randomization of the study design, which is the nature of this type of study design. However, it was not practicable in implementing the alcohol sale restriction for randomization. In addition, we could not identify any appropriate independent control groups with similar population characteristics near the two towns under study. Therefore, we used interrupted time series analysis design with ARIMA modeling to compare measures before and after the intervention. We believe that by using this “before-after” quasi-experimental design, the variations of population characteristics between groups could be reduced rather than increased.

In conclusion, this study provided evidence that the restriction of selling high strength alcohol has contributed to the significant reduction in alcohol-related hospitalizations and ED attendances in Fitzroy Crossing and Halls Creek for the whole study period although the effectiveness of intervention varied in the two towns. The use of interrupted time series analysis with ARIMA modeling and applying AAFs enabled us to estimate changes due to alcohol more accurately. The WA AAFs can be modified by using other jurisdictional specific alcohol consumption prevalence and applied to similar studies.

## Author Contributions

WS, LJ, and JX designed the study and contributed to draft and revisions. WS and LJ analyzed this work. PS and GA provided comments and contributed to drafts and revisions. All the authors confirmed the last version.

### Conflict of Interest Statement

The authors declare that the research was conducted in the absence of any commercial or financial relationships that could be construed as a potential conflict of interest.
